# Enhancing the Performance of Tangential Flow Microfiltration for Bioreactor Clarification

**DOI:** 10.3390/membranes15030078

**Published:** 2025-03-03

**Authors:** Amir Hossein Mostafavi, Liang-Kai Chu, Xianghong Qian, John Paul Smelko, Da Zhang, Andrew Zydney, Sumith Ranil Wickramasinghe

**Affiliations:** 1 Ralph E. Martin Department of Chemical Engineering, University of Arkansas, Fayetteville, AR 72701, USA; am223@uark.edu (A.H.M.); dzhang@benitec.com (D.Z.); 2 Department of Chemical Engineering, The Pennsylvania State University, University Park, PA 16802, USA; liang-kai.chu@merck.com (L.-K.C.); zydney@engr.psu.edu (A.Z.); 3 Department of Biomedical Engineering, University of Arkansas, Fayetteville, AR 72701, USA; xqian@uark.edu; 4 Biogen, 5000 Davis Drive, Research Triangle Park, NC 27709, USA; john.smelko@biogen.com

**Keywords:** bioreactor harvesting, clarification, continuous biomanufacturing, fouling, hydrocyclone, membrane morphology, microfiltration

## Abstract

Tangential flow microfiltration is easily adapted for batch and continuous bioreactor clarification. The permeate can be introduced directly to the subsequent capture step. However, the commercial use of tangential flow filtration (TFF) is limited by membrane fouling, leading to a compromised performance. Here, we explored the possibility of reducing membrane fouling by integrating a hydrocyclone as the primary clarification operation. The overflow from the hydrocyclone was introduced directly as the feed to the microfiltration module. Chinese hamster ovary cells were used as the feed stream to investigate the feasibility of this integrated process. A range of cell viabilities from 0% (cell lysate) to 96% were investigated. The cell densities ranged from 0.9 to 10 million cells per mL. Two commercially available hollow fiber microfiltration membranes were used, an essentially symmetric membrane and a reverse asymmetric membrane where the more open support structure faced the feed stream. The reverse asymmetric membrane was more resistant to fouling in the absence of an integrated hydrocyclone. Integrating a hydrocyclone led to a reduction in the flux decline for the symmetric membrane, but did not affect the performance of the reverse asymmetric membrane. The careful choice of membrane morphology and pore size is important when designing an integrated process.

## 1. Introduction

Biotechnology manufacturing processes make use of cell culture operations to grow cells that produce the desired therapeutic (e.g., monoclonal antibodies, virus vectors, etc.). Bioreactor clarification operations are used to remove the unwanted particulate matter (cells, cell debris, etc.) from the growth medium that contains the product of interest. Typically, manufacturing processes are run in batch mode.

However, there is significant interest in the development of continuous biomanufacturing processes [1]. The major advantages of continuous manufacturing processes are lower capital and operating costs. This results from the more efficient utilization of the process equipment, a smaller footprint and equipment size, and a higher productivity due to less equipment downtime [2]. However, in the biopharmaceutical industry, the cost of goods has not been the dominant driver, explaining the slower development of continuous biomanufacturing processes. This has begun to change with the development of biosimilars and the desire to enable greater US-based manufacturing to reduce the reliance on overseas supply chains. Additionally, continuous biomanufacturing can lead to improved product quality resulting from the much shorter residence times and steady state operation.

Most of the advances in continuous biomanufacturing have focused on upstream processes [3]. Significant progress has been made on the development of continuous cell culture processes, with media added continuously while the product is removed in what is commonly referred to as a perfusion bioreactor. Cost analyses indicate that higher cell densities obtained in continuous cell culture operations lead to higher production rates and lower costs [4]. Unlike batch operations, where the bioreactor is clarified at the end of the cell culture operation, the product needs to be removed continuously while retaining the cells in perfusion operations. Consequently, some type of cell retention device must be integrated with the bioreactor. A variety of cell retention devices have been explored with different methods of action, such as filtration, sedimentation, centrifugation, ultrasonic fixation, and dielectrophoretic exclusion [5]. Filtration-based methods are of particular interest since they can effectively remove both whole cells and relatively small cell debris, depending upon the pore size of the filter. Here, we focused on tangential flow filtration (TFF), as it can easily be incorporated into batch and continuous manufacturing processes.

TFF has many advantages over other bioreactor clarification operations for both batch and continuous processing [6]. If an appropriate-pore-size membrane is used, e.g., 0.22 or 0.45 mm, the permeate is sufficiently “clean” to be directly fed to the subsequent capture step (most commonly Protein A chromatography for monoclonal antibody production). However, the high cell density in state-of-the-art bioreactors often causes membrane fouling, leading to product rejection and a low filtrate flux, compromising the viability of TFF. Further, especially for emerging therapeutics such as virus vectors, cell lysis is often required, as the product of interest is not excreted into the growth medium. TFF for the clarification of cell lysates is extremely challenging due to rapid membrane fouling.

TFF can easily be incorporated as a cell retention device in perfusion operations. However, high cell densities tend to lead to higher rates of fouling. Higher rates of fouling invariably lead to product sieving losses during operation, especially over extended periods of time. Three methods may be used to minimize fouling during TFF. The membrane structure can be modified. Da et al. [7,8] indicated that the use of a reverse asymmetric membrane where the more open pore structure faces the feed stream and the barrier layer faces the permeate can lead to higher productivity during batch operation. Alternatively, one can modify the TFF operating conditions. For example, alternating tangential filtration (ATF), in which the direction of the feed flow is alternated in a cyclic fashion, can minimize fouling, but the operating filtrate flux in these systems is typically <3 L m^−2^ h^−1^ [9]; thus, large membrane areas are required for commercial manufacturing operations.

A third approach for enhancing the performance of TFF that is appropriate for both batch and continuous operations is to integrate TFF as a secondary clarification step with another unit operation used to reduce the load of particulate matter (cells and cell debris) before performing TFF. In perfusion processes, the primary clarification operation must be amenable for continuous operation with the recycling of the retained cells. Thus, depth filtration, which is widely used for initial clarification, is not viable in this application. The incorporation of an appropriate continuous primary clarification step would reduce the particulate load in the feed to the TFF step, minimizing fouling and the associated reductions in product recovery during extended operation of the perfusion bioreactor. Modular chemical process intensification, where two unit operations are combined to achieve an integrated system with a higher performance than each of the individual unit operations, is of growing interest in the biotechnology industry [10,11]. Here, we investigated for the first time the use of a hydrocyclone as the primary clarification operation in combination with TFF as the secondary clarification operation.

**Figure 1:**
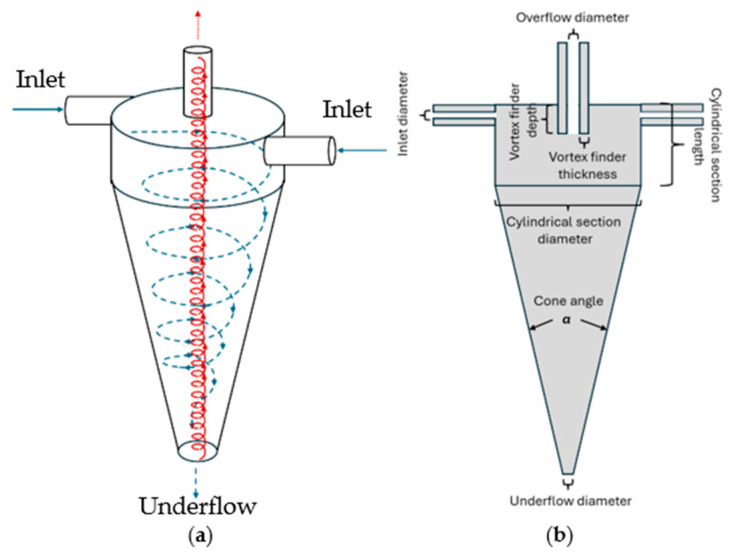
Schematic of a hydrocyclone; (**a**) flow pattern and (**b**) nomenclature for relevant components.

Hydrocyclones have been used for many years for particle separations from liquid streams in the mineral-processing, chemical, and petrochemical industries. They can be easily scaled up and used in continuous manufacturing and have low operating costs [12,13]. The feed stream is introduced at the top of the hydrocyclone, as shown in Figure 1a. There are two exits from the hydrocyclone: the underflow, which contains the larger (higher-density) particulate matter, and the overflow, which contains a low concentration of smaller (lower-density) particles. The larger and higher-density particles move towards the wall due to centrifugal and drag forces as well as the pressure gradient. They then move downwards and into the underflow. The smaller and lower-density particles move upwards and exit with the overflow [14]. The degree of separation of the particles into the underflow depends on the rate of centrifugal sedimentation. While the principle of separation is the same as in classical centrifugation, the hydrocyclone has no moving parts, eliminating many of the challenges of large-scale continuous centrifuges.

Several investigators have examined the use of a hydrocyclone alone for mammalian cell separations [15,16]. These studies indicate that, for CHO cells, a separation efficiency in the overflow versus underflow as high as 99% can be obtained with cell viabilities as high as 90% at pressure drops of up to 3 bar. Hydrocyclones have also been used as a cell retention device for perfusion bioreactors where similar cell viabilities were achieved. Ni et al. [13] provided a review of the important design parameters.

Hydrocyclones are ideally suited for the separation of particles 10 mm or larger, but they are much less effective at removing smaller cell debris. Particles of a given size will reach an equilibrium radial position where the outward terminal settling velocity equals the inward radial velocity of the liquid (see Figure 1a). Particles with an equilibrium position that coincides with the locus of zero liquid vertical velocity have an equal probability of leaving via the underflow or overflow. This is known as the cut size. Reducing the cut size by optimizing the various geometric parameters will lead to higher feed pressures. However, high feed pressures are undesirable for perfusion bioreactors, as they will adversely affect cell viability. Thus, a secondary clarification operation will be required after the hydrocyclone to obtain a product stream suitable for subsequent downstream processing operations.

Syed et al. [17] used a hydrocyclone for primary cell separation, with a spiral microchannel used for secondary clarification, as the cell retention device for a perfusion bioreactor. They indicated up to 75% separation of cells by the hydrocyclone with <5% loss in cell viability. The spiral microchannel system removed more than 90% of the remaining particulate matter; thus, some type of additional clarification would likely still be needed. In addition, scaling up the spiral microchannel device is likely to be challenging. He et al. [18] investigated in detail the viability loss of CHO cells for a continuous cell culture using hydrocyclones. They indicate that cell viability decreases with an increase in the hydrocyclone cone angle (see Figure 1b) and inlet velocity due to the increased pressure drop to which the cells are subjected. The highest separation efficiencies obtained by He et al. [18] were <90%, although there was no discussion of how the required secondary clarification would be performed.

The current study provides the first analysis of using a hydrocyclone in combination with TFF in order to improve the performance of the TFF operation. The hydrocyclone reduces the load of particulate matter in the feed stream to the microfiltration unit, significantly reducing the rate of fouling and increasing the performance of the TFF unit. Two different hydrocyclones were fabricated using 3D printing based on literature designs. Two different TFF modules were investigated, with one containing an essentially symmetric membrane and one having a reverse asymmetric membrane. The effects of the different membrane morphologies are also discussed. The results provide insights into the development of an integrated hydrocyclone–TFF bioreactor clarification process with applications in batch and continuous bioprocessing operations.

## 2. Materials and Methods

### 2.1. Materials

The reagents used in this study were biotechnology grade or higher if not specified. FreeStyle™ CHO-S cells were obtained from ThermoFisher (Waltham, MA, USA). CHOgro^®^ Expression Media and the poloxamer 188 (Pluronic) solution, 10% *w*/*v* in cell-culture-grade water, were purchased from Mirus Bio (Madison, WI, USA). L-glutamine, TRITON™ X-100, and sodium hydroxide were purchased from VWR (Radnor, PA, USA). Trypan blue was purchased from Sigma-Aldrich (Burlington, MA, USA).

All the rubber tubing and peristaltic pumps used to deliver fluids were purchased from Masterflex (Vernon Hills, IL, USA). The water used in this work was obtained from a ThermoFisher 18 MΩ Barnstead Smart2pure system (Schwerte, Germany). An electronic scale (PL602-S) was purchased from Mettler Toledo (Columbus, OH, USA). Two hollow fiber microfiltration modules were custom manufactured for this work by Asahi Kasei Bioprocess (Glenview, Il, USA). The BioOptimal™ MF-SL filter had a membrane surface area of 0.00041 m^2^, with a pore size of 0.4 μm on the permeate side and 40 μm on the feed side. The module is unique in that the more open support structure faces the feed, unlike conventional microfiltration modules in which the size-selective “skin” faces the feed. The UJP module has a membrane surface area of 0.00032 m^2^. The barrier layer, which faces the feed stream, has a nominal pore size of 0.65 μm, as provided by the manufacturer. While there is variation in the average pore size from the inside to the outside of the fiber, the membrane is much more symmetric in structure. Both hollow fiber membranes were manufactured by a wet spinning process. The membrane materials were polyvinylidene-fluoride for the UJP and polysulfone for the BioOptimal MF-SL filters. The hydrocyclones were fabricated by 3D printing as described below.

### 2.2. Cell Culture

FreeStyle™ CHO-S cells were cultured using flasks in a shaker incubator at 37 °C and 125 rpm. A humidified atmosphere consisting of 8% CO_2_ was used. Cell titers of up to 25 million cells/mL were obtained using CHOgro^®^ Expression media supplemented with 8 mM L-glutamine and 0.3% pluronic. First, CHO cells were thawed and then passaged after a few days repeatedly to obtain the desired concentration. The cell density was evaluated under a microscope using a hemacytometer with trypan blue exclusion dye used to identify the number of dead cells. The trypan blue exclusion assay is based on the principle that live cells have intact cell membranes that will exclude the dye, while dead cells will turn blue. The cell sample was mixed with dye and then visually examined. Viable cells appeared clear while nonviable cells appeared blue.

The cell viability was determined by Equation (1):

(1) $$Cell\;Viability\;\left(\%\right)=\frac{N_{v}}{N_{t}}\times 100$$

where $N_{v}$ and $N_{t}$ are the number of viable and total cells, respectively.

### 2.3. Hydrocyclone Designs

There have been numerous studies that have considered the design of hydrocyclones [19]. Several geometrical and operational factors should be considered, among which the inlet and outlet diameters, cone angle, vortex finder depth and thickness, inlet pressure and velocity, and concentration of particulate matter are important [12,13]. The inlet and outlet diameters and ratios primarily influence the velocity of the fluid and the pressure drop. For mammalian cells, these two factors are important, as high pressures lead to a reduction in cell viability.

**Figure 2:**
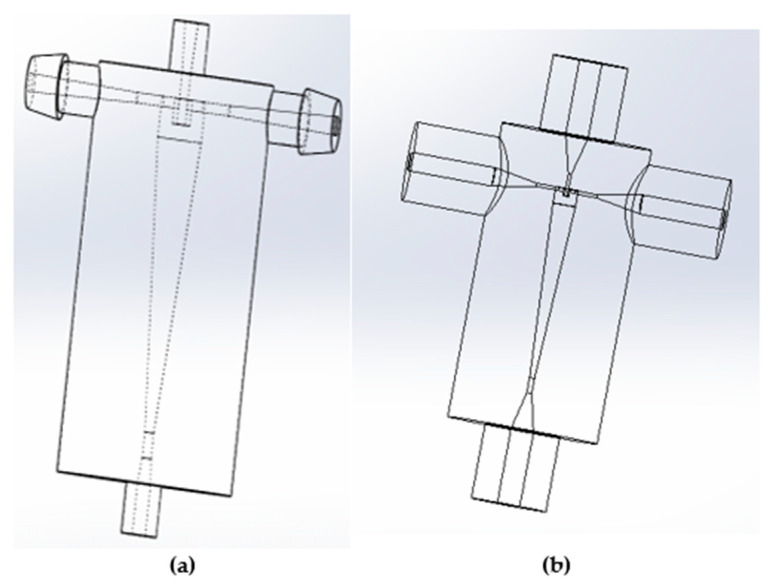
CAD drawings of hydrocyclones in SolidWorks. (**a**) Design I and (**b**) design II.

The hydrocyclones in this study were made using Fromlab 3-D printers (Somerville, MA, USA) using gray and clear resins (Figure 2). The designs were based on the hydrocyclones used in previous bioreactor clarification studies [17,18]. Table 1 shows the design parameters. Design I (larger hydrocyclone) is based on the work by He et al. [18], while design II is based on the work by Syed et al. [17]. As can be seen, design I is simpler and has the same inlet and outlet diameters. Both hydrocyclones are based on the Bradley hydrocyclone.

Table 2 compares important design ratios for the two hydrocyclones to the ratios provided by Bradley [20]. The Bradley hydrocyclones have been shown to provide high separation efficiencies [19]. As can be seen, both hydrocyclones have elements of Bradley’s optimized ratios. The SolidWorks PDM 2023 software (Cleverbridge, Inc., Chicago, IL, USA) was used to design the hydrocyclones prior to printing, as shown in Figure 2.

### 2.4. Experimental Setup and Procedure

**Figure 3:**
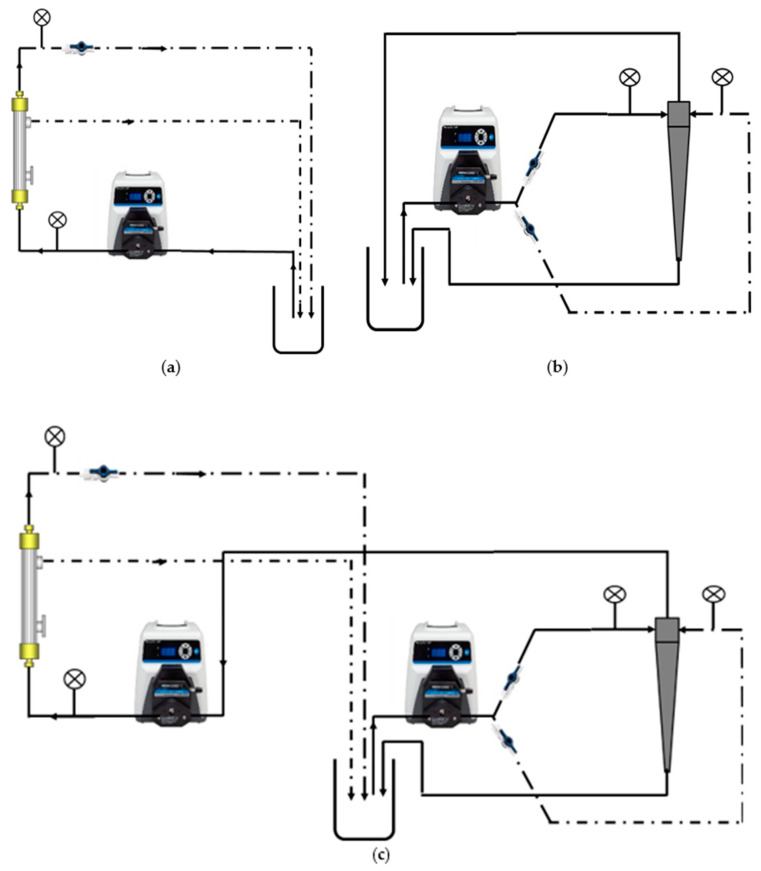
Experimental setups used here (**a**) TFF, (**b**) hydrocyclone, and (**c**) integrated hydrocyclone–TFF.

Three different clarification operations were considered: TFF only, hydrocyclone only, and an integrated hydrocyclone and TFF process, as shown in Figure 3.

Before starting each experiment, the hydrocyclone was washed with 10% bleach followed by DI water. Then, the supplemented media was circulated through the system prior to adding the cell suspension. The TFF filters were also cleaned, regenerated, and washed with PBS, a NaOH/bleach solution, and then DI water, in that order. For the hydrocyclones, prior to testing with the cell suspension, the underflow DI water split ratio was determined using Equation (2).

(2) $$Split\;ratio\;\left(\%\right)=\frac{Q_{U}}{Q_{F}}\times 100$$

where $Q_{U}$ and $Q_{F}$ are the underflow rate and the feed flow rate, respectively. Various cell densities were investigated. We compare our result to the work of He et al. [18] and Syed et al. [17]. He et al. determined the separation efficiency as

(3) $$E\;\left(\%\right)=\left(\frac{Q_{U}C_{U}}{Q_{U}C_{U}+Q_{O}C_{O}}\right)\times 100$$

where $C_{U}$ and $C_{O}$ are the cell concentrations in the underflow and overflow, respectively. Syed et al. defined the separation efficiency differently as

(4) $$\;E\;\left(\%\right)=\left(1-\frac{C_{O}}{C_{F}}\right)\times 100$$

where $C_{F}$ is the cell concentration in the feed. The results obtained here were compared to the respective literature data based on these definitions, given by Equations (3) and (4).

### 2.5. Bioreactor Harvesting

Prior to use, the microfiltration modules were flushed with 1 L of DI water. The DI water was pumped through the fiber lumen with the permeate line open, enabling water to pass through the retentate and permeate flow paths. The same procedure was used for the UJP and BioOptimal MF SL filters. This filter preparation was not expected to have any effect on filter performance.

The membrane was regenerated after use according to the following protocol. The filter was first flushed with 1–2 L of DI water at room temperature to remove cells and weakly bound debris. The feed was pumped through the fiber lumen and the permeate line was opened, allowing water to pass through the retentate and permeate lines. Next, the membrane was back-flushed (fluid pumped from outside the fibers through the membrane to the fiber lumen) using about 100 mL of 0.5 N NaOH with 3000 ppm NaClO at room temperature. The module was then forward-flushed (i.e., fluid flows from fiber lumen through the membrane to the shell side) using 0.5 N NaOH with 3000 ppm NaClO at 30 mL/min for 30 min at room temperature. Using the same solution composition, the module was back-flushed at 30 mL/min for 30 min. The last two steps were repeated 3–4 times. The filter was then stored overnight in 0.5 N NaOH with 3000 ppm NaClO. Finally, the next day, the filter was flushed (flow from the feed side to the retentate side) with 1–7 L of water until the pH was neutralized. The nominal water flux for the BioOptimal MF-SL was >21,000 L m^−2^ h^−1^. For the UJP filter, the nominal water flux was 1200 L m^−2^ h^−1^. Both fluxes were at a TMP of 0.1 MPa and a temperature of 25 °C.

Two sets of TFF experiments were run. The first series of experiments involved running the UJP and BioOptimal MF-SL filters at a wall shear rate of 2000 s^−1^ (feed flow rate of 16 mL min^−1^ for the UJP filter and 32 mL min^−1^ for the BioOptimal MF-SL filter) in concentration mode. A wall shear rate of 2000 s^−1^ was chosen based on the manufacturer’s recommendations and previous studies. The experiments were run in batch concentration mode. The cell density was 9 million/mL and the feed volume was 50 mL. The initial transmembrane pressure (TMP) was 3.4 kPa (0.5 psi) and 13 kPa (2 psi) for the BioOptimal MF-SL and UJP filters, respectively. It is essential that the BioOptimal is run at a low TMP in order to prevent compaction of the stabilized cake layer within the more open membrane pore structure that faces the feed stream. These TMP values resulted in an initial permeate flux of 1100 L m^−2^ h^−1^ for both modules. Experiments were also run at different cell viabilities using the BioOptimal MF-SL filter under the same experimental conditions. The cell viability was decreased by reducing the rate of nutrient addition. The experiments were designed to investigate the effect of the reverse asymmetric membrane structure on flux decline.

The second set of TFF experiments was run with and without an integrated hydrocyclone. These experiments were run in total recycle mode. The feed flow rate to the hyrocyclone was 50 mL min^−1^. The overflow from the hydrocyclone was introduced to the TFF module at a feed flow rate of 10 mL min^−1^, corresponding to wall shear rates of 620 s^−1^ and 1280 s^−1^ for the BioOptimal MF-SL and UJP filters, respectively. The initial TMP for TFF only (without the hydrocyclone) was 3.7 ± 0.14 kPa (0.54 ± 0.02 psi) for the BioOptimal MF-SL and 12.4 ± 1.3 kPa (1.8 ± 0.19 psi) for the UJP filter. For the integrated system, the TMP was 4.0 ± 0.41 kPa (0.58 ± 0.06) and 12.1 ± 0.83 kPa (1.76 ± 0.12 psi) for the BioOptimal and UJP modules, respectively.

Additional experiments were conducted using the BioOptimal MF-SL filter in ATF mode. A Repligen (Waltham, MA, USA) alternating tangential flow-2 (ATF-2) filtration system was used for perfusion system setup. The setup used a diaphragm pump to both withdraw and return the cell suspension to the bioreactor. The perfusion rate was 300 mL min^−1^, i.e., 300 mL of fluid was pumped in and out of the bioreactor in 1 min. Although no frequency was set, considering that the displacement of one exchange is 120 mL, about 20 s was estimated for 1 displacement (120 mL). The frequency was controlled by the ATF flow rate. The ATF flow rate is the most important parameter. The set points for the pH, temperature, and DO were 7, 37 °C, and 40%. A Sartorius (Göttingen, Germany) BioStat A plus with a starting cell density of 1 million viable cells/mL in a 1.5 L working volume was used. Perfusion was initiated one day after inoculation at 1 reactor volume per day (RV/day). The perfusion rate was then increased to 1.5 RV/day when the cell density approached a value of 10 million viable cells/mL. After that, the perfusion rate was maintained at 1.5 RV/day. The perfusion run was performed for 14 days.

## 3. Results

### 3.1. Membrane Structure

**Figure 4:**
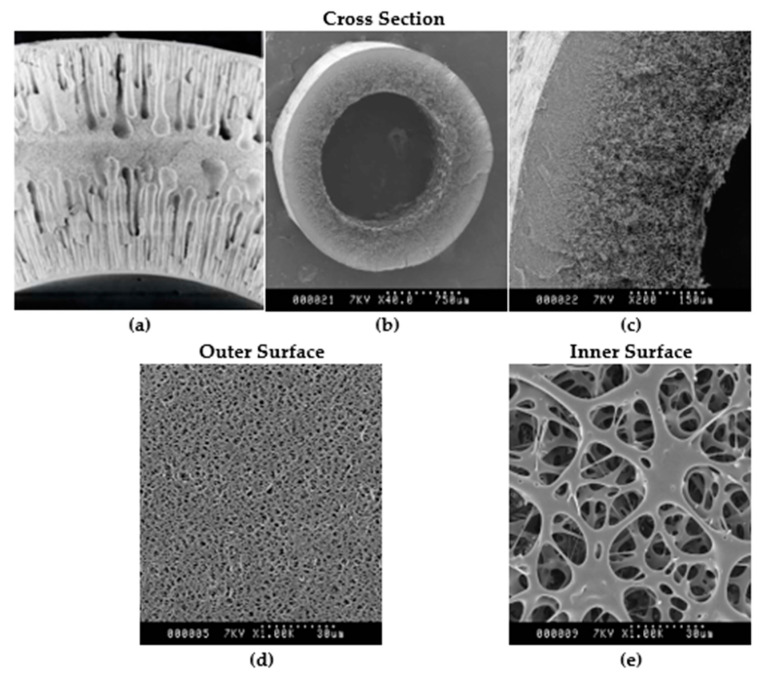
SEM images of (**a**) UJP cross section, with inside diameter of 1.1 mm, courtesy of Asahi Kasei Corporation; (**b**–**e**) BioOptimal MF-SL, with inside diameter of 1.4 mm; and (**b**,**c**) cross section showing clear asymmetric structure, (**d**) outside surface, and (**e**) inside surface, courtesy of Asahi Kasei Bioprocess.

Figure 4 gives SEM images of the membranes in the UJP and BioOptimal MF-SL filters. These images were obtained from the manufacturer. As can be seen from Figure 4a, which gives a cross-sectional image of the UJP membrane, the membrane is rather symmetric. Figure 4b–e give various images of the BioOptimal MF-SL membrane. The membrane has a much more open pore structure in the fiber lumen which is in contact with the feed. The barrier layer is located at the outer fiber surface. As can be seen for the UJP membrane, the surface porosity appears similar though at the middle of the fiber the porosity is much less. In the case of the asymmetric BioOptimal membrane, the inner surface has a much higher porosity than the outer surface.

In our earlier work [7,8], we showed that the more open structure stabilizes a highly permeable cake layer that acts as a depth filter. The stabilized cake layer removes particles that would otherwise foul the barrier layer.

### 3.2. Effect of Cell Viability on TFF

**Figure 5:**
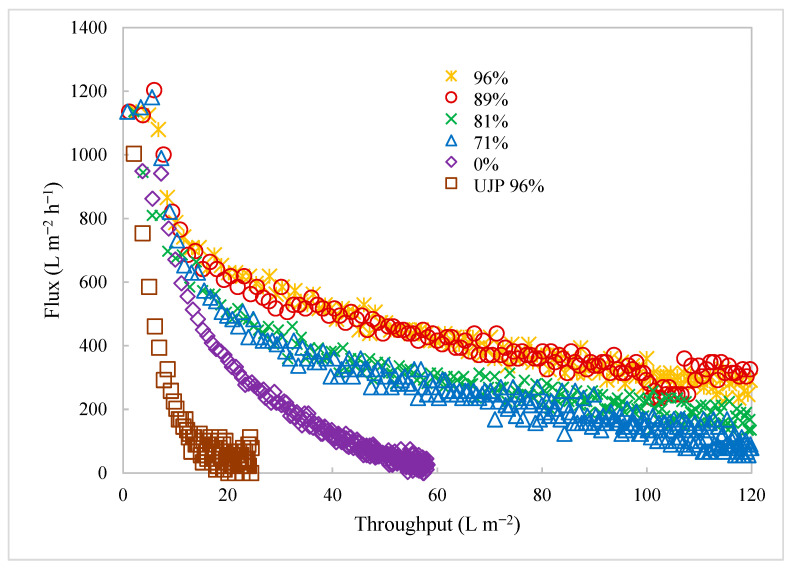
Flux versus productivity for BioOptimal MF-SL and UJP filters under a range of feed conditions. Percentages in the legend represent different cell viabilities. All results are for BioOptimal unless noted in the legend.

Figure 5 gives the permeate flux versus throughput (permeate recovered per membrane surface area) for the first set of TFF experiments. Data are included for the BioOptimal MF-SL and UJP modules at a wall shear rate of 2000 s^−1^ run in batch concentration mode. The cell viability was over 96% for the UJP. The lower-viability experiments for the BioOptimal MF-SL used cell suspensions that were partially lysed to reduce the cell viability. The initial TMP was chosen to give the same initial permeate flux, 1110 L m^−2^ h^−1^. Since, industrially, TFF is run under constant flux conditions, the performance of the two modules was compared at the same flux. The results indicated a rapid flux decline for the UJP module as the contents of the feed reservoir became concentrated. The BioOptimal MF-SL displayed a much higher throughput during the batch concentration.

Figure 5 also gives the flux versus throughput results for the BioOptimal MF-SL for cell viabilities ranging from 0 to 89%. Given the very low throughput for the UJP membrane, even at cell viabilities of over 96%, it was not used to examine the effect of cell viability on the throughput. As can be seen, there was a rapid decrease in the throughput with reduced viability. When all the cells were dead, with 0% viability representative of a cell lysate, the throughput was at its lowest. Dead cells tend to lyse, leading to a large amount of small particulate matter that can easily deposit on and within the membrane. These results indicate that, although the reverse asymmetric structure of the BioOptimal MF-SL membrane can lead to much higher throughputs than observed for the UJP membrane, low-viability feed streams still cause extensive fouling, which would lead to significant challenges in the application of these membranes for cell recycle and primary clarification during long-term continuous perfusion operations.

### 3.3. Hydrocyclone Performance

**Figure 6:**
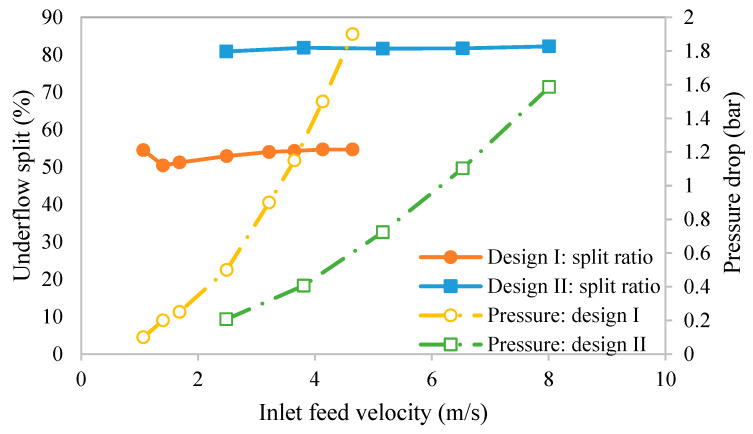
Variation in underflow split ratio and pressure drop with inlet feed velocity.

Before using the hydrocyclones as part of the integrated clarification process, initial testing was conducted to determine if the performance of the hydrocyclones developed in this work were similar to the ones designed by He et al. [18] and Syed et al. [17]. Figure 6 gives the variation in the inlet velocity with the underflow DI water split ratio, as defined by Equation (2). The right-hand *y*-axis gives the pressure drop. As can be seen, for both designs, the percentage of the total feed that exits via the underflow (split ratio) was nearly independent of the inlet feed velocity over the range of conditions examined in Figure 6. However, the underflow split ratio was much higher for design II. The split ratio is affected by a number of factors. The important factors here are the overflow and underflow outlet ratios compared to the size of the inlet or cylindrical sections. Figure 1b and Table 1 give the important dimensions of the hydrocyclones. A larger underflow-to-inlet diameter ratio compared to the overflow-to inlet diameter ratio will lead to a higher split through the underflow. On the other hand, a larger vortex finder depth and a higher-pressure drop will lead to a higher underflow split ratio. Here, although design I had a larger vortex finder depth and displayed a higher pressure drop, the influence of the underflow-to-inlet diameter dominated, leading to the higher split ratio for design II. These results indicate that it was the combined effects of the relevant variables that led to the observed split ratio. The inlet pressure results also show that design I experienced a greater pressure increase with increasing velocity. The higher pressure drop was due to the steeper cone angle and lower outlet/inlet ratios.

**Figure 7:**
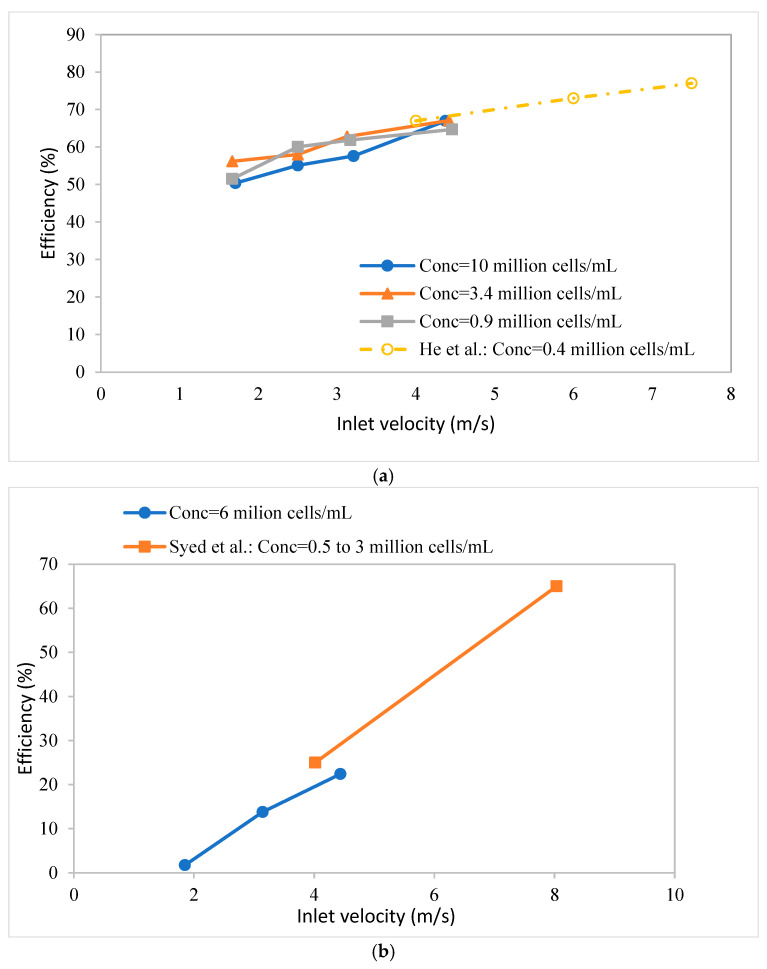
Separation efficiency versus inlet velocity. (**a**) Comparison of efficiency of hydrocyclone design 1 with results of He et al. [18]; (**b**) comparison of efficiency of design II with results of Syed et al. [17].

Figure 7 gives the separation efficiencies of the two hydrocyclones tested here. Figure 7a compares the efficiency of design I with the results obtained by He et al. [18], while Figure 7b compares the efficiency of design II with the results obtained by Syed et al. [17]. In both cases, the separation efficiency is plotted against the inlet feed velocity. Figure 7a,b indicate that, even though lower feed flow rates (and consequently, pressure drops) were used here, the results are in good agreement with those from previous studies using the same hydrocyclone designs. Over the range of cell densities investigated here, it appears that cell density has little effect on the separation efficiency. It is worth noting that the cell viability dropped significantly to less than 90% at higher operating velocities (similar to the literature studies), likely due to the very high pressures under these conditions. Therefore, our experiments focused on the performance at lower inlet velocities than reported in the literature. Specifically, for design I, the viability loss was more significant due to the higher normal stress (pressure), as shown in Figure 6.

Cell viability depends on the feed flow rate (shear stress to which the cells are subjected) and the time for which they are subjected to a given shear stress. With an increasing run time, the viability drops. However, since these runs were conducted under non-sterile conditions, the cell viability could also drop due to bacterial infection. For design 1, Figure 7a shows that, at all flow rates, the viability dropped over a 2 h period to below 90%. The decrease in viability was much greater at higher flow rates. For design II, only at the highest feed flow rate investigated did the viability drop to below 90% after 2 h.

### 3.4. ATF with BioOptimal MF SL Microfilter

**Figure 8:**
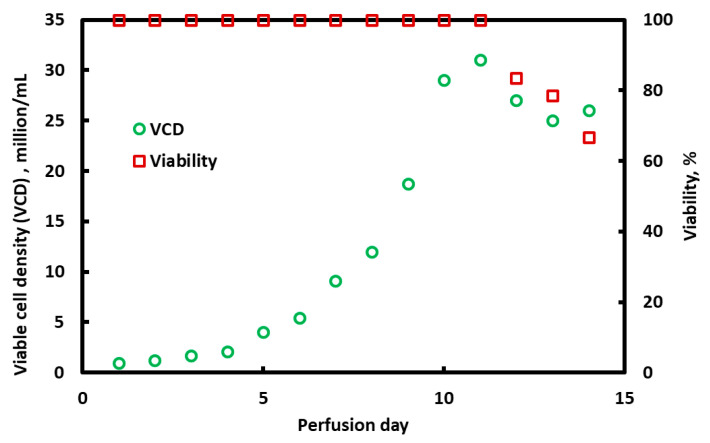
Variation in viable cell density and cell viability over a 14-day perfusion experiment. A BioOptimal MF SL microfilter was run in ATF mode as the cell retention device.

Figure 8 provides the results for the experiment conducted with the BioOptimal MF SL filter in ATF mode. The viable cell density (left-hand-side *y*-axis) and cell viability (right-hand-side *y*-axis) are plotted against the perfusion time in days. The run was conducted for 14 days. The perfusion rate was 1 vessel volume (1.5 L per day, or 1 mL/min) for the first 5 days (i.e., below 10 million viable cells/mL) and then 1.5 vessel volumes (2.25 L per day, or 1.5 mL/min) for the rest of the run (cell densities larger than 10 million viable cells/mL). The average permeate flux was about 152 L m^−2^ h^−1^ for the first 5 days and 229 L m^−2^ h^−1^ for the remainder of the run. No fouling occurred and the TMP was below 0.5 psi (2.5 kPa) throughout the 14 days of operation.

### 3.5. Integrated Hydrocyclone TFF

**Figure 9:**
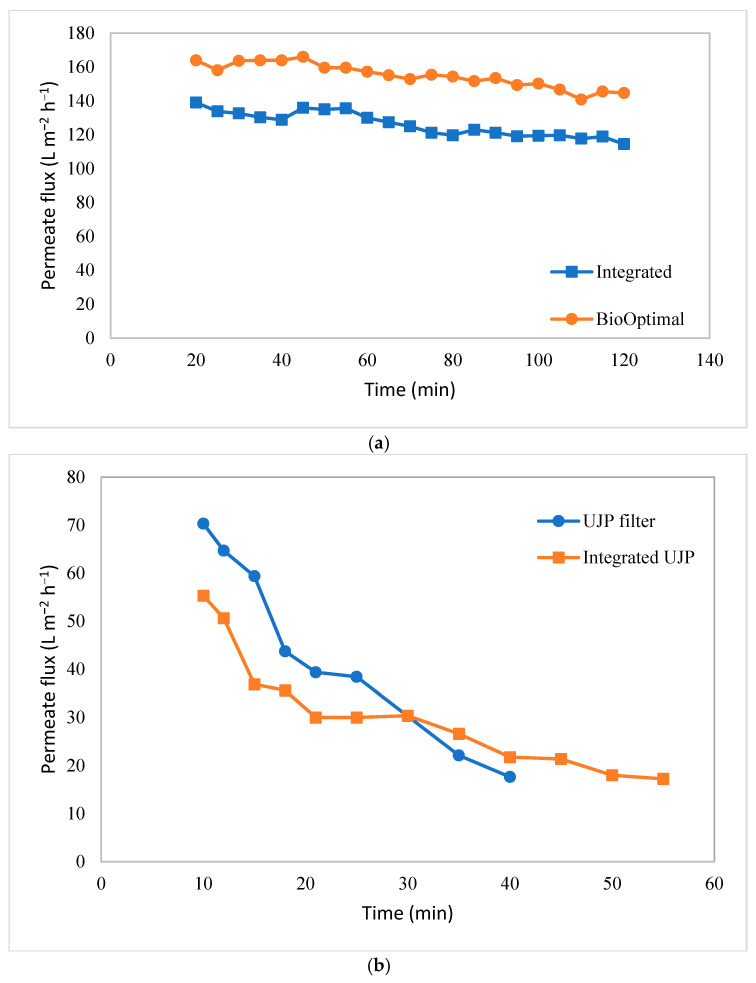
Variation in permeate flux with time: (**a**) BioOptimal and hydrocyclone–BioOptimal system and (**b**) UJP and hydrocylone–UJP system. Data for the integrated systems were obtained with the same module used for the TFF alone, but after cleaning/regeneration.

As shown in Figure 3c, an integrated hydrocyclone–TFF system was tested where the overflow from the hydrocyclone was used as the feed to the TFF module. Figure 9 gives the variation in the permeate flux with time. Figure 9a gives the results for the BioOptimal filter by itself and for the same module integrated with the hydrocyclone. Figure 9b gives the analogous results for the UJP module. As can be seen, the BioOptimal displayed a much higher permeate flux than the UJP module with and without an integrated hydrocyclone. Further, the rate of flux decline over a 2 h period was much greater for the UJP module. The cell viability was 97% at the start of the 2 h run and remained at approximately 97% at the end of the run for both modules. The much more stable flux for the BioOptimal module is similar to the results shown in Figure 5 and indicates the benefit of using a reverse asymmetric membrane in this application.

Figure 9a suggests that the flux decline for the BioOptimal was similar with and without an integrated hydrocyclone. The small difference in flux for the two runs was likely due to a small loss in the permeability of the module. The module was tested without the hydrocyclone; it was cleaned/regenerated at the end of the experiment and then used again for the integrated process. The permeability of the regenerated membrane was within 90% of the initial water flux through the clean module, but the small decrease in permeability likely caused the slightly lower flux for the integrated process. These results indicate that, for the BioOptimal module, an integrated hydrocyclone had little effect on membrane fouling under the operating conditions investigated, likely due to the effectiveness of the open pore structure in protecting the size-selective skin on the outer surface of the membrane from fouling by cells.

The results for the UJP module are shown in Figure 9b. Again, the initial flux was a little lower for the integrated process due to the reduction in the permeability of the regenerated module. However, the rate of flux decline for the integrated clarification process was significantly less than that for the UJP module alone. In this case, the ratio of the flux at t = 40 min to that at t = 10 min was 0.25 for the UJP module alone compared to 0.39 for the same module, but after primary clarification using the hydrocyclone. The net result is that the flux for the integrated hydrocyclone–TFF process was greater than that for the TFF alone for t > 30 min. These results suggest that, by using a hydrocyclone for initial clarification, the rate of flux decline due to membrane fouling can be reduced for conventional TFF microfiltration membranes in which the barrier layer faces the feed.

## 4. Discussion

TFF is a very attractive unit operation for clarification of cell culture media in perfusion bioreactors, as the permeate can typically be introduced directly to the first capture chromatography operation without any additional clarification. Furthermore, TFF can be easily adapted for both batch and continuous clarification. However, membrane fouling is a serious limitation that restricts the practical use of TFF. Figure 5 indicates that, for traditional TFF membranes, where the barrier layer faces the feed stream, rapid flux decline occurs due to absorption of foulants and deposition of rejected species directly on or within the barrier layer of the membrane. As shown in Figure 5 and in our earlier studies [7,8], the use of a reverse asymmetric membrane can be beneficial in this application, as the support structure helps stabilize a highly permeable layer of rejected particulate matter. Foulants are removed by this dynamic membrane, thus protecting the barrier layer that faces the permeate stream from membrane fouling. However, as seen in Figure 5, as the cell viability decreases, cell debris will cause significant fouling even of these reverse asymmetric membranes, leading to a more rapid flux decline and the need to replace the membrane module to enable long-term continuous operations. In a continuous process, the flux would be much lower, but must be maintained for up to 2 months.

Here, for the first time, we combined a hydrocyclone with TFF for clarification of a CHO cell culture. The hydrocyclone provides the primary clarification operation, significantly reducing the particulate concentration in the feed to the TFF module. For traditional TFF membranes where the barrier layer faces the feed, reducing the foulant load in the feed leads to more stable fluxes over a longer time (see Figure 9b). Note that these experiments were not run under sterile conditions, which limited the duration of the integrated clarification process. It is likely that, if the integrated hydrocyclone–TFF experiments were conducted under sterile conditions for several days, the more stable permeate flux for the integrated process would be more significant when compared to the TFF operation alone.

In contrast, at least over a 2 h period, it appears that the integrated process offers no measurable advantage when using the BioOptimal module for the second clarification step. A similar result was observed in parallel studies, where a BioOptimal module was used in ATF mode for 2 weeks. In this experiment, the permeate flux and the rate of medium addition to the bioreactor were matched: 152 L m^−2^ h^−1^ for the first 5 days of operation and then 229 L m^−2^h^−1^ for days 6 to 14 of operations. Both the traditional TFF membranes and the reverse asymmetric membrane showed no flux decline over a 2-week period. ATF has been proposed as another way to suppress membrane fouling. This method typically uses slightly larger-inner-diameter hollow fiber membranes, although any TFF membrane could potentially be used. ATF makes use of altered fluid mechanics and operation at a very low filtrate flux to suppress membrane fouling. A diaphragm pump is used that removes and returns the feed through the same port [21,22]. While feed flow reversal leads to suppression of fouling, a disadvantage of the process is that the permeate fluxes are low, resulting in very large membrane surface areas for large-scale commercial operation.

The results obtained here indicate that, for a reverse asymmetric membrane that is more resistant to flux decline, the benefits of an integrated process are likely to be greater at a low cell viability or for extremely long run times, e.g., 2 months. An integrated process may be well suited for continuous biomanufacturing operations, particularly when using conventional TFF membranes in which the size-selective layer faces the cell culture media. Further investigations under sterile conditions for much longer run times will be required in order to determine the effectiveness of integrating a hydrocyclone as the primary clarification step prior to TFF. Nevertheless, the membrane choice will be important (pore size, morphology, asymmetric structure, etc.) for the design and implementation of an optimized two-step clarification process incorporating both a hydrocyclone and a TFF membrane unit.

## 5. Conclusions

Developing methods to improve the performance of TFF for bioreactor clarification is highly desirable, as it can be adapted for both batch and continuous operations. Furthermore, the careful selection of the membrane pore size will enable the direct introduction of the permeate to the subsequent capture step in the downstream process. There have been many attempts to overcome membrane fouling, which is a major limitation that leads to product sieving losses, low permeate fluxes, and the need to periodically replace/regenerate the membrane. Here, the feasibility of integrating a hydrocyclone with TFF was investigated for the first time. Like TFF, a hydrocyclone can also be adapted for batch and continuous operations. The overflow from the hydrocyclone is depleted of large particulate matter, thus reducing the fouling potential of the feed in the TFF module. Care will be needed when integrating these two unit operations, as the overflow rate will determine the feed flow rate and wall shear rate for the TFF module. Furthermore, selecting an appropriate membrane morphology will be important. The results obtained here indicate that, for reverse asymmetric membranes that are much more resistant to fouling, integrating a hydrocyclone may be of minimal benefit, while significant improvements in the TFF performance may be possible when using more conventional asymmetric microfiltration membranes.

## Figures and Tables

**Table 1:** Design parameters for hydrocyclones fabricated as part of this study. Design 1 is based on He et al. [18]. Design II is based on Syed et al. [17].

Parameter	Dimensions (mm) (Design I)	Dimensions (mm) (Design II)
Cylindrical section diameter	10	3
Overflow diameter	2	0.4
Underflow diameter	2	0.65
Inlet diameter	2	0.413
Length of the cylindrical section	8	2.16
Cone length	57.2	23.16
Vortex-finder depth	5	1
Vortex-finder thickness	1	0.2–0.4

**Table 2:** Comparison of geometric ratios for the hydrocyclones in comparison with Bradley’s ratios.

Ratio	Design I	Design II	Bradley’s Ratio
Inlet/cylindrical section diameter	0.2	0.133	0.133
Overflow/cylindrical section diameter	0.2	0.133	0.2
Vortex finder depth/cylindrical section diameter	0.5	0.33	0.33
Cone length/cylindrical section diameter	5.7	7.7	6.58

## Data Availability

The original contributions presented in this study are included in the article. Further inquiries can be directed to the corresponding authors.
